# Cyclin-Dependent Kinase 1 Is Essential for Muscle Regeneration and Overload Muscle Fiber Hypertrophy

**DOI:** 10.3389/fcell.2020.564581

**Published:** 2020-10-14

**Authors:** Yutaka Kobayashi, Tomoyuki Tanaka, Mieradilli Mulati, Hiroki Ochi, Shingo Sato, Philipp Kaldis, Toshitaka Yoshii, Atsushi Okawa, Hiroyuki Inose

**Affiliations:** ^1^Department of Orthopaedics, Graduate School, Tokyo Medical and Dental University, Tokyo, Japan; ^2^Department of Rehabilitation for Movement Functions, National Rehabilitation Center for Persons with Disabilities, Research Institute, Tokorozawa, Japan; ^3^Center for Innovative Cancer Treatment, Tokyo Medical and Dental University Hospital, Tokyo, Japan; ^4^Department of Clinical Sciences, Lund University, Clinical Research Centre, Malmö, Sweden; ^5^Department of Orthopedic and Trauma Research, Graduate School, Tokyo Medical and Dental University, Tokyo, Japan

**Keywords:** Cdk1, muscle regeneration, satellite cell, cell cycle, sarcopenia, frail, muscle injury

## Abstract

Satellite cell proliferation is an essential step in proper skeletal muscle development and muscle regeneration. However, the mechanisms regulating satellite cell proliferation are relatively unknown compared to the knowledge associated with the differentiation of satellite cells. Moreover, it is still unclear whether overload muscle fiber hypertrophy is dependent on satellite cell proliferation. In general, cell proliferation is regulated by the activity of cell cycle regulators, such as cyclins and cyclin-dependent kinases (CDKs). Despite recent reports on the function of CDKs and CDK inhibitors in satellite cells, the physiological role of Cdk1 in satellite cell proliferation remains unknown. Herein, we demonstrate that Cdk1 regulates satellite cell proliferation, muscle regeneration, and muscle fiber hypertrophy. Cdk1 is highly expressed in myoblasts and is downregulated upon myoblast differentiation. Inhibition of CDK1 activity inhibits myoblast proliferation. Deletion of *Cdk1* in satellite cells leads to inhibition of muscle recovery after muscle injury due to reduced satellite cell proliferation *in vivo*. Finally, we provide direct evidence that Cdk1 expression in satellite cells is essential for overload muscle fiber hypertrophy *in vivo*. Collectively, our results demonstrate that Cdk1 is essential for myoblast proliferation, muscle regeneration, and muscle fiber hypertrophy. These findings could help to develop treatments for refractory muscle injuries and muscle atrophy, such as sarcopenia.

## Introduction

Satellite cells, also known as muscle stem cells, play a significant role in myogenesis and muscle regeneration. Mammalian adult skeletal muscle is a relatively stable tissue that undergoes little cell death or nuclear fission during its normal life cycle (Chargé and Rudnicki, 2004; Stephenson and Kojouharov, 2018). Accordingly, satellite cells are in a quiescent cell cycle state under normal conditions (Relaix and Zammit, 2012). In response to stimulation, such as muscle injury, muscle regenerates via the activation and proliferation of satellite cells to form a pool of myoblasts (Relaix and Zammit, 2012). Myoblasts then differentiate and fuse to provide the myonuclei required to repair or replace damaged myofibers, the contractile units of skeletal muscle (Yin et al., 2013). Accordingly, understanding the regulatory mechanism of skeletal muscle cell differentiation and proliferation is essential for developing strategies to treat muscle disorders such as muscle injury and atrophy. Recent progress in molecular biological research has uncovered various regulatory processes of myogenesis and muscle regeneration. Central to this regulation are transcription factors; Myf5, MyoD, Myogenin, and Mrf4, which are important for differentiation of skeletal muscle cells (Zammit, 2017). However, the mechanisms regulating satellite cell proliferation are relatively unknown compared to the knowledge associated with activation and differentiation of satellite cells. Moreover, it is still unclear whether overload muscle fiber hypertrophy is dependent on satellite cell proliferation, since conflicting data has been published (Mccarthy et al., 2011; Egner et al., 2016; Goh and Millay, 2017; Fukada, 2018; Fukuda et al., 2019). This lack of understanding of the mechanisms of satellite cell proliferation, muscle regeneration, and muscle fiber hypertrophy could be one of the reasons why so far no effective drugs have been discovered for sarcopenia and muscle injury.

Cellular proliferation is regulated by cell cycle regulators, such as cyclins and cyclin-dependent kinases (CDKs) (Lim and Kaldis, 2013). CDKs are a group of kinases consisting of 20 members in mammals, which play critical roles in cell cycle control, transcription, and development (Satyanarayana and Kaldis, 2009; Saito et al., 2016; Takahashi et al., 2018). Previous studies have revealed the role of cell cycle regulators in muscle biology (Marroncelli et al., 2018; White et al., 2018). For example, p16^Ink4a^, a CDK inhibitor, accelerated the entry of satellite cells into a senescent state (Zhu et al., 2019), CDKN1c (p57^Kip2^), a CDK inhibitor, coordinates the balance between proliferation and growth arrest in satellite cells (Mademtzoglou et al., 2018), and the overexpression of CDK4 in human myoblasts was useful for immortalization and associated with the maintenance of cell proliferative capacity (Zhu et al., 2007; Shiomi et al., 2011). However, the physiological roles of CDK1 in myogenesis and muscle regeneration have not been extensively studied. Moreover, no CDKs have been found to regulate muscle regeneration through *in vivo* cell-specific loss-of-function experiments.

In this study, we generated satellite cell-specific *Cdk1* knockout mice to investigate the regulatory role of Cdk1 in satellite cell proliferation, muscle regeneration, and muscle fiber hypertrophy.

## Experimental Procedures

### Animals

*Cdk1f/f* mice and the Pax7^CreER^ mouse line have been described previously (Murphy et al., 2011; Diril et al., 2012). We crossed Pax7^CreER^ mice with *Cdk1f/f* mice to obtain Pax7^CreER^ /*Cdk1f/f* mice. We then crossed Pax7^CreER^ /*Cdk1f/f* mice with *Cdk1f/f* mice, and their offspring Pax7^CreER^ /*Cdk1f/f* female mice were used for the experiment. Pax7^CreER^/*Cdk1f/f* mice were administered either vehicle (corn oil) or tamoxifen (100 mg/kg body weight) for five consecutive days by intraperitoneal (IP) injection. After a 1-week washout period, mice were used for experiments. All mice were maintained under standard conditions with food and water available ad libitum under a 12 h light/dark cycle. All animal experiments were performed with the approval of the Animal Study Committee of Tokyo Medical and Dental University and conformed to relevant guidelines and laws.

### Genotyping

PCR genotyping of Cdk1, wild-type, FLOX, and null alleles, primers Pr1, Pr2, and Pr3 were used at 1 μM final concentration as previously described (Diril et al., 2012). Thirty-five PCR cycles with 30 s denaturation at 94°C, 30 s annealing at 68°C, and 30 s extension at 72°C were performed to amplify different alleles of Cdk1, resulting in a band of 159 bp (Cdk1WT), 255 bp (Cdk1FLOX), or 389 bp (Cdk1NULL). PCR genotyping of Pax7^creER^ was performed as previously described (Murphy et al., 2011). Primer sequences are shown in Supplementary Table S1.

### Cell Culture

Cells were purchased from the Riken Cell Bank (Tsukuba, Japan). C2C12 cells were maintained in Dulbecco’s Modified Eagle’s Medium containing 4.5 g/L glucose (DMEM-high glucose; Sigma), 100 units/mL penicillin, 10 μg/mL streptomycin and 10% fetal bovine serum (FBS; Sigma) at 37°C in a humidified atmosphere of 5% CO_2_. To induce differentiation, cells were cultured in differentiation medium (DMEM containing 2% horse serum) with or without 10 μM RO-3306 (Sigma) to inhibit Cdk1 activity. The cells were replenished with fresh differentiation medium every 3 days. For cell counting, we counted the total number of cells in the visual field of four different regions.

### Quantitative Real-Time PCR Analysis

RNA from cultured cells was extracted using TRIzol reagent (Invitrogen). Reverse transcription was performed using the High-Capacity cDNA Reverse Transcription Kit (Applied Biosystems) according to the manufacturer’s instructions. We performed quantitative analysis of gene expression using the Mx3000p qPCR system (Agilent Technologies). *Gapdh* expression was used as an internal control. Primer sequences are shown in Supplementary Table S2.

### Western Blotting Analysis

For immunological detection, 20 μg of cell lysate was separated via SDS-PAGE (7.5–10% Tris gel). After the proteins were blotted onto a PVDF membrane, the membrane was incubated with the PVDF blocking reagent Can Get Signal (TOYOBO). Proteins were probed with primary antibodies against CDK1 (MBL) and GAPDH (MBL) and secondary antibodies were detected through autoradiography using enhanced chemiluminescence (ECL Plus, General Electric Healthcare).

### Cardiotoxin-Induced Muscle Injury

Eight-week old female mice were anesthetized with ketamine (100 mg/kg) and xylazine (10 mg/kg) and 100 μL of 10 μM Cardiotoxin (LATOXAN) or PBS were injected into the tibialis anterior muscles. The injured muscles were collected at 3 and 14 days after injury and processed for histochemistry. All mice were euthanized using a high dose of ketamine and xylazine then the tibialis anterior muscles were removed, quickly frozen in liquid nitrogen-cooled 2-methylbutane, and stored at –80°C.

### Histology of the Tibialis Anterior Muscle

Ten microgram transverse sections of the tibialis anterior muscles were cut with a cryostat (CM 300; Leica Japan, Tokyo, Japan) and kept at –80°C. The sections were stained with hematoxylin and eosin (H&E). The slides were evaluated under light microscopy, and microphotographs were taken with a digital camera (Olympus AX70; Olympus, Tokyo, Japan) attached to a microscope (Olympus BX51; Olympus).

### Muscle Mass Measurement

Dissected tibialis anterior muscles were weighed with a precision balance (Sartorius, sensitivity 0.1 mg). The muscle mass data were normalized relative to the PBS-injected contralateral muscle in the same mouse.

### *In situ* Muscle Force Measurement

The mice were anesthetized 14 days after the cardiotoxin injection and set in the supine position. Tibialis anterior muscle was then surgically exposed, and the distal tendon of the muscle was tied with a 6–0 suture line. After cutting the tendon, the suture line was used to connect the tibialis anterior muscle to the lever arm of the force transducer (TB-653T; Nihon Koden, Tokyo, Japan) and recorded with a sensor interface (Power lab; AD Instruments Japan, Nagoya, Japan) using software (Power lab software; AD Instruments Japan). The hindlimb was stabilized by attaching the patella tendon to a fixed post using a needle insert. The proximal end of tibialis anterior muscle was stimulated with an electrostimulator (Neuropack μ; Nihon Koden) at 1 Hz (twitch) or 50 Hz (tetanus), and the maximum strength was recorded.

### Immunohistochemistry and Immunofluorescence

The cultured C2C12 cells in 24-well plates were fixed with 4% paraformaldehyde for 10 minutes. 0.3% Triton X-100 was used for permeabilization. The cells were then blocked in 10% normal goat serum (Jackson ImmunoReserch). After blocking, the cells were incubated with primary antibodies at room temperature for 1 hour. Subsequently, the secondary fluorescent antibodies were added to the cells at room temperature for 1 hour followed by DAPI (Nacalai) staining. Frozen samples were embedded in 4% carboxymethyl cellulose (CMC) sodium (Leica Microsystems A/S) and fixed with 4% paraformaldehyde for 10 minutes. After the blocking step with Mouse-on-Mouse Blocking Reagent (Vector Laboratories), 10 μm frozen tissue sections were immunostained overnight with primary antibodies at 4°C. After incubation with secondary antibodies followed by DAPI staining, slides were mounted in Fluorescence Mounting Medium (DAKO) and stored at 4°C in the dark. Detection by microscopy was performed on a BZ-9000 fluorescence microscope (Keyence, Osaka, Japan), and composite images were created using ImageJ (National Institutes of Health, Bethesda, MD). The primary antibodies used were anti-Cdk1 (Abcam, 1:100), anti-Pax7 (Developmental Studies Hybridoma Bank, 1:40), anti-Laminin (Abcam, 1:100), anti-Myogenin (Abcam, 1:100), anti-Myosin heavy chain (Developmental Studies Hybridoma Bank, 1:2), and anti-Ki67 (Abcam, 1:100) and goat anti-rabbit IgG, goat anti-mouse IgG1, and goat anti-mouse IgG2a cross-absorbed secondary antibodies (Invitrogen, 1:800) were applied.

### Fusion Index

Fusion indices were calculated as previously described (Veliça and Bunce, 2011). In brief, fusion index was determined by dividing the nuclei numbers in fused myotubes (Myosin heavy chain positive cells with 2 or more nuclei) by the total number of nuclei. The images were captured randomly at 3 different spots.

### TUNEL Staining

Satellite cell apoptosis in 12-week-old mouse tibialis anterior muscle was examined via TUNEL assays. TUNEL assays were performed with the ApopTag system (Millipore) according to the manufacturer’s instructions with modifications (Mulati et al., 2020). Briefly, after applying the anti-digoxigenin conjugate, a tyramide signal amplification system (PerkinElmer) was used to detect fluorescent signals.

### Cross-Sectional Area (CSA) Measurement

At least 10 images were acquired manually at ×20 of magnification of a muscle cryosection immunostained with a rabbit anti-laminin antibody. We used Open-CSAM, an ImageJ macro, to perform a semi-automated analysis of CSA on skeletal muscle. If necessary, at the end of the automated measurement, manual correction was performed using ImageJ. The selection tools in the ROI Manager were used to remove “false” myofibers created by the automation, and the “freehand selections” tool was used for hand-drawing “lacking” myofibers missed by the automation. To calculate mean fiber CSAs, at least 1,000 fibers were measured for each sample.

### Overload Surgery

Mice were anesthetized with ketamine (100 mg/kg) and xylazine (10 mg/kg). A midline incision was made in the skin on the hindlimbs, and the distal tendons of tibialis anterior muscle was transected. Overload of the extensor digitorum longus muscle was achieved by excising approximately two-thirds of the distal end of the tibialis anterior muscle. The incision was closed using 6–0 nylon suture. Ablations were performed unilaterally, and all animals were subjected to 2 weeks of synergist ablation (OL+). Mice on which no surgery served as controls (OL−). After 2 weeks of OL, all mice were euthanized using a high dose of ketamine and xylazine then the extensor digitorum longus muscles were removed, quickly frozen in liquid nitrogen-cooled 2-methylbutane, and stored at –80°C. Muscle weights were normalized against body weights.

### Statistics

All data are presented as the means ± SD (*n* ≥ 3). We performed statistical analysis using Student’s *t*-test, and *P* < 0.05 was considered statistically significant.

## Results

### Cdk1 Expression Is Decreased During Myoblast Differentiation

**FIGURE 1:**
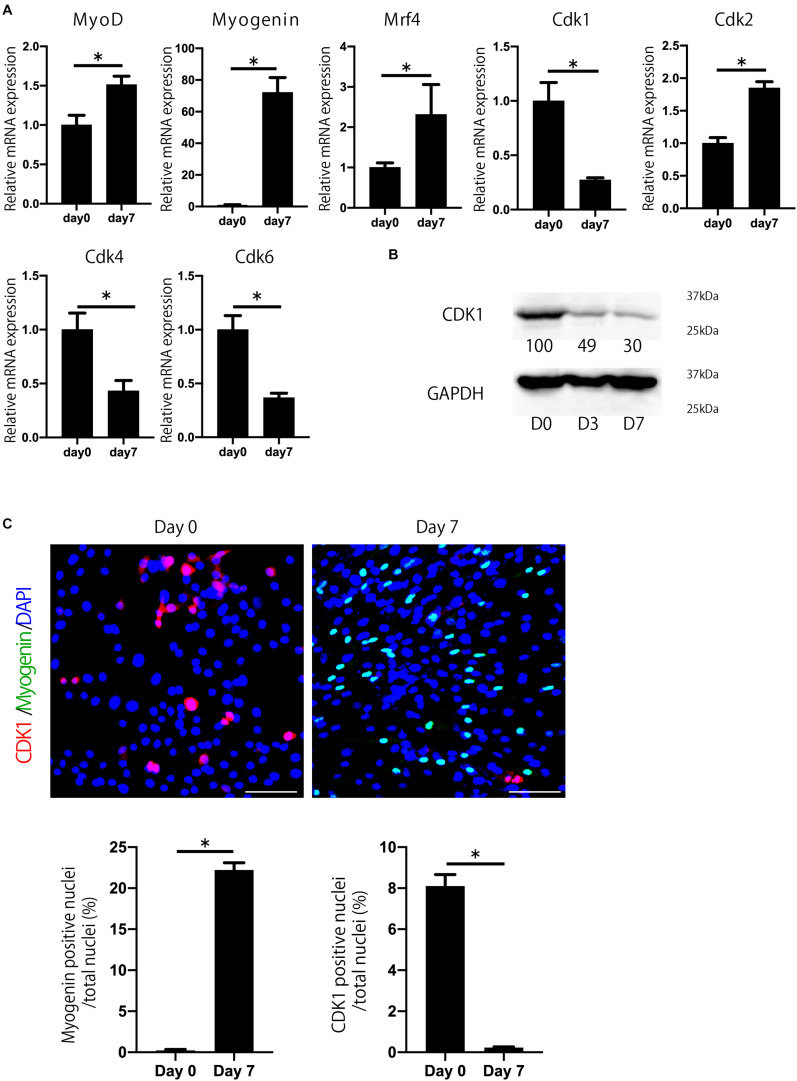
Cdk1 expression is decreased during myoblast differentiation. **(A)** The change in expression of *Cdks* during C2C12 myoblasts was examined by qPCR. *Cdk1* expression was more prominently downregulated than other *Cdks*. *P < 0.05 vs. day 0. **(B)** Changes in the expression of CDK1 during myoblast differentiation as determined at the protein level using western blot analysis. The band intensities representing CDK1 expression level were quantitated with reference to GAPDH control bands. The intensities of protein bands were quantitated using image J. The value of day 0 was set at 100 and the quantified values were displayed below the bands. The protein levels of CDK1 decreased during the course of myoblast differentiation. **(C)** Immunohistochemistry analysis of CDK1 (red) and MYOGENIN (green) of C2C12 cells. Nuclei are stained with DAPI (blue). The expression of CDK1 was observed on day 0, but it was decreased on day 7 when the cells were cultured in myogenic differentiation medium. In contrast, the expression of myogenin, a marker of myogenesis, was low on day 0, but abundant on day 7. Scale bars: 100 μm. ^∗^*P* < 0.05.

To investigate which Cdks are involved in myogenesis and muscle regeneration, we determined the expression of Cdks during myoblast differentiation. We used C2C12 myoblasts, which were induced to differentiate into myotubes as described (Yaffe and Saxel, 1977). C2C12 cells express several Cdks, including *Cdk1*, *Cdk2*, *Cdk4*, and *Cdk6* during myoblast differentiation (Figure 1A). Among them, we focused on *Cdk1* because its expression was most downregulated during myoblast differentiation (Figures 1A,B), whereas *Cdk2* was upregulated. Furthermore, we performed immunostaining to confirm CDK1 protein expression during myoblast differentiation. In this experiment, to investigate the difference in CDK1 expression between the proliferating and differentiating states, we defined day 0 as the day before cell density became confluent, when myoblast proliferation was still taking place. Then, the next day (day 1), after the cells became confluent, the growth medium was changed to the differentiation medium. MYOGENIN, a marker of muscle differentiation, was slightly expressed on day0, but was significantly increased on day 7, suggesting that our experiments were properly conducted (Figure 1C). On the other hand, CDK1 was abundantly expressed on day 0, but significantly decreased on day 7 (Figure 1C), mirroring our previous experiments (Figures 1A,B).

### Cdk1 Plays an Important Role in Myoblast Proliferation

**FIGURE 2:**
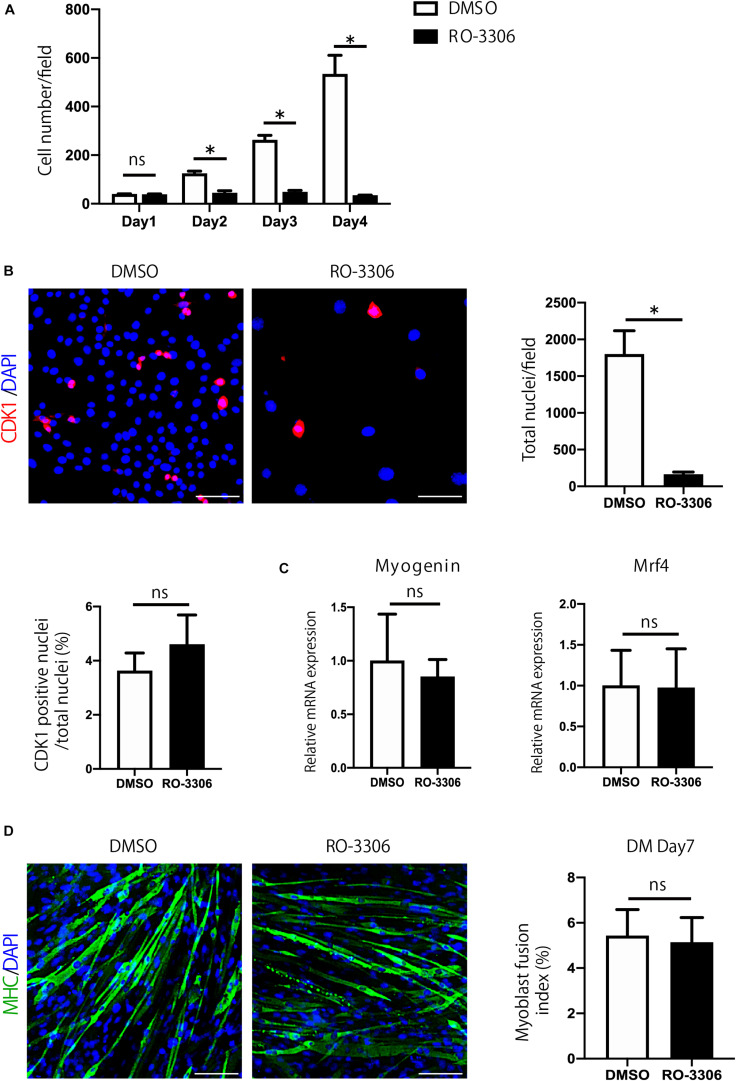
Cdk1 plays an important role in myoblast proliferation. **(A)** The relative number of C2C12 myoblasts treated with vehicle or RO-3306 was counted for 4 days. Note that RO-3306 treatment of C2C12 myoblasts impaired proliferation compared to vehicle-treated cells. ^∗^*P* < 0.05 vs. controls. **(B)** Immunohistochemistry analysis of CDK1 (red) of C2C12 cells. Nuclei are stained with DAPI (blue). Inhibition of CDK1 activity reduced the total number of cells, but the ratio of CDK1-positive cells to total cells was not significantly different between RO-3306 and vehicle groups. Scale bars: 100 μm. ^∗^*P* < 0.05. n.s., not significant. **(C)** The inhibition of CDK1 activity did not affect myoblast differentiation as judged *Myogenin* and *Mrf4* mRNA levels. n.s., not significant. **(D)** Immunohistochemistry analysis of myosin heavy chain (green) of C2C12 cells. Nuclei are stained with DAPI (blue). The fusion index, a marker of muscle differentiation, was not significantly different from that of the vehicle group, even when CDK1 activity was inhibited by RO-3306 treatment. MHC, myosin heavy chain. DM, differentiation medium. n.s., not significant. Scale bars: 100 μm.

Next, to examine the importance of Cdk1 in myoblast proliferation, we treated C2C12 cells with RO-3306, a specific inhibitor of CDK1 activity (Vassilev, 2006). Compared with the proliferation of vehicle-treated cells, the proliferation of C2C12 cells was significantly impaired by RO-3306 treatment (Figure 2A). Inhibition of CDK1 activity reduced the total number of cells compared to the vehicle group, but the ratio of CDK1-positive cells to total cells was not significantly different between RO-3306 and vehicle groups (Figure 2B). These results were consistent with the results in other types of cells (Saito et al., 2016; Takahashi et al., 2018). Since a decrease in proliferation can affect differentiation, we tested whether inhibiting CDK1 activity would affect myoblast differentiation. However, according to the results of qPCR, the expression of Myogenin and Mrf4, markers of muscle differentiation, did not differ significantly from the vehicle group with RO-3306 treatment (Figure 2C). Furthermore, immunostaining for the myosin heavy chain indicated that the fusion index, another marker of muscle differentiation, was not significantly different from that of the vehicle group, even when CDK1 activity was suppressed by RO-3306 treatment (Figure 2D). Taken together, these results indicate that CDK1 is more important for proliferation than for differentiation in myoblasts.

### Satellite Cell-Specific *Cdk1*-Knockout Mice Display Impairment in Muscle Regeneration

**FIGURE 3:**
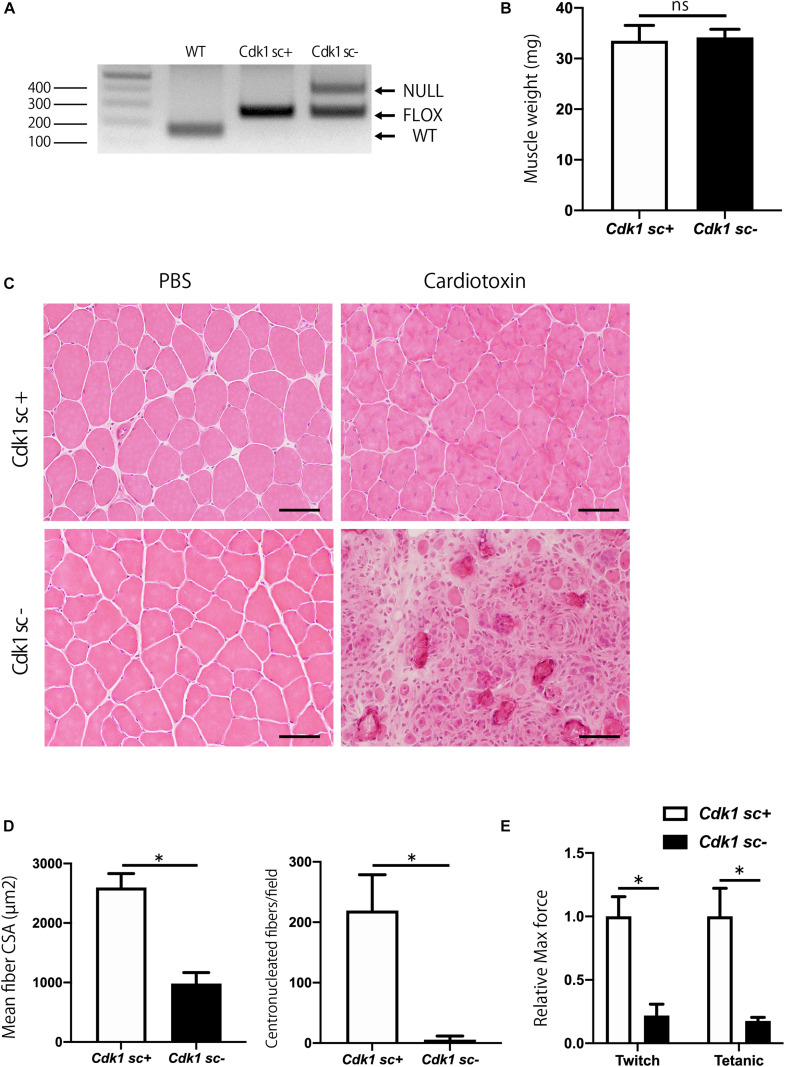
Impairment of muscle regeneration in satellite cell-specific *Cdk1*-knockout mice. **(A)** PCR genotyping demonstrated the presence of null allele in the tibialis anterior muscle of *Cdk1sc*– mice. **(B)** Muscle weight showed no difference in tibialis anterior muscles of *Cdk1sc+* and *Cdk1sc*– mice. **(C)** Histologic evaluation of muscle recovery 2 weeks after cardiotoxin injection. H&E images of tibialis anterior muscles. Muscle regeneration was impaired and inflammatory cell infiltration was prevalent in *Cdk1sc*– mice. Scale bars: 50 μm. **(D)** Mean fiber cross-sectional area (CSA) and centronucleated fibers were decreased in *Cdk1sc*– mice after cardiotoxin injection. ^∗^*P* < 0.05. **(E)** Relative twitch and tetanic force were decreased in tibialis anterior muscles of *Cdk1sc*– mice after cardiotoxin injection. The force of cardiotoxin-treated leg of each sample was normalized to the respective uninjured control leg. We set the relative force in control mice as the standard for comparison. ^∗^*P* < 0.05 vs. controls.

To investigate the role of Cdk1 in muscle regeneration *in vivo*, we generated conditional satellite cell-specific *Cdk1* knockout mice to investigate the role of Cdk1 in muscle regeneration *in vivo*. Because *Cdk1*-null mice exhibit early embryonic lethality (Diril et al., 2012) we used a conditional approach. To achieve this, we crossed *Cdk1f/f* mice with transgenic mice expressing Cre recombinase under the control of the Pax7 promoter (hereafter, “Pax7^CreER^ mice”) to generate Pax7^CreER^/*Cdk1f/f* mice (hereafter, “*Cdk1sc+*”). These mutant mice allow for the specific and inducible deletion of *Cdk1* in satellite cells upon tamoxifen treatment with all other cell types retaining CDK1 expression. We administered vehicle or tamoxifen by IP injection to 8 weeks of age female control mice for five consecutive days to delete *Cdk1* effectively in satellite cells (hereafter, “*Cdk1sc*− *“*). As a first approach, we extracted DNA from the tibialis anterior muscles of 12-week-old *Cdk1sc+* and *Cdk1sc−* mice and performed genotyping to investigate whether Cdk1 was deleted in satellite cells. We found a null allele in the muscle tissue of *Cdk1sc−* mice, indicating that *Cdk1* was deleted in PAX7-expressing satellite cells in the tibialis anterior muscle (Figure 3A). The additional visible floxed Cdk1 allele is most likely due to contamination of PAX7- cells. We then examined the muscle weight of *Cdk1sc+* and *Cdk1sc*− mice at the age of 12 weeks. There was no significant difference in tibialis anterior muscle weight between the two groups (Figure 3B). Next, we investigated whether the deletion of *Cdk1* affects muscle regeneration. In control mice, muscle regeneration following intramuscular cardiotoxin injection was evident 14 days after injury, as demonstrated by the presence of myofibers with centrally located nuclei (Figure 3C). However, while uninjured tamoxifen-treated muscles did not show any signs of atrophy, muscle regeneration was severely impaired in *Cdk1sc*− mice (Figure 3C, lower right panel). Despite including small regenerated muscle fibers and normal uninjured muscle fibers together in the measurements, the mean fiber cross sectional area (CSA) was significantly decreased in the *Cdk1sc*− mice (Figure 3D). The number of centronucleated fibers, indicating regenerating muscle fibers, was significantly reduced in the *Cdk1sc−* mice (Figure 3D). We also checked the functional outcomes of deteriorated muscle regeneration in *Cdk1sc*− mice. As expected, *ex vivo* force measurement of tibialis anterior muscles resulted in both maximum twitch force and tetanic force significantly decreased in *Cdk1sc*− mice compared with control mice after muscle regeneration (Figure 3E). Collectively, these results suggested that Cdk1 was important in muscle regeneration *in vivo*.

### Satellite Cell Proliferation Is Decreased in Satellite Cell Specific *Cdk1* Knockout Mice

**FIGURE 4:**
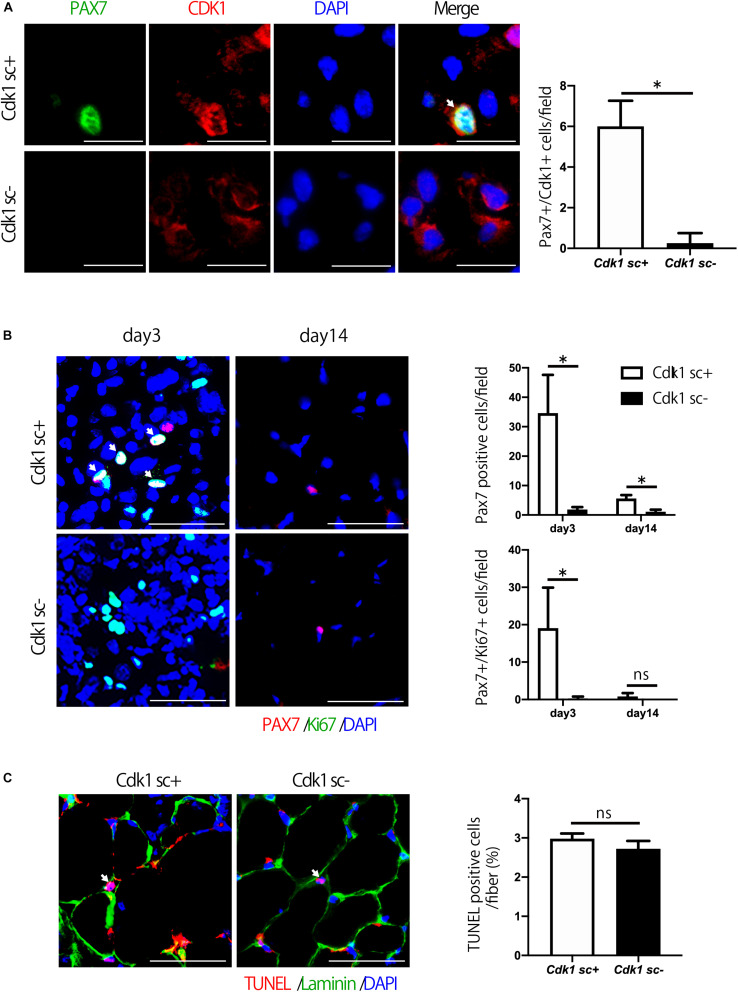
Satellite cell proliferation is decreased in satellite cell-specific *Cdk1* knockout mice. **(A)** Immunohistochemistry analysis of CDK1 (red) and PAX7 (green) of 3-month-old mouse tibialis anterior muscles. Nuclei are stained with DAPI (blue). These images show fewer CDK1 and PAX7 double-positive satellite cells (white arrows) in *Cdk1sc*– mice than in *Cdk1sc+* mice 3 days after cardiotoxin injection. Scale bars: 20 μm. ^∗^*P* < 0.05. **(B)** Immunohistochemistry analysis of Ki-67 (green) and PAX7 (red) of mouse tibialis anterior muscles. DAPI (Blue) was used to stain the nuclei. These images show fewer Ki-67 and PAX7 double-positive satellite cells (white arrows) in the *Cdk1sc*– sections than in the *Cdk1sc+* sections at 3 days after cardiotoxin injection. Scale bars: 50 μm. ^∗^*P* < 0.05. n.s., not significant. **(C)** TUNEL assays were performed in 3-month-old *Cdk1sc+* and *Cdk1sc*– mice 3 days after cardiotoxin injection. No difference in apoptotic TUNEL positive cells (white arrows) was detected between the two groups. Scale bars: 50 μm. n.s., not significant.

We then aimed to investigate the molecular mechanism behind the impairment of muscle regeneration observed in *Cdk1sc*− mice. Firstly, we performed immunostaining for CDK1 and PAX7 in muscle tissue to confirm that CDK1 expression was absent in satellite cells of *Cdk1sc*− mice. Indeed, we found that in *Cdk1sc*− mice, CDK1 expression was significantly down-regulated in satellite cells (Figure 4A). Then, to test whether decreased muscle regeneration was caused by altered proliferation and/or apoptosis, we performed immunohistochemistry for PAX7 and Ki67, and TUNEL staining. While immunohistochemistry revealed many PAX7- and Ki67- double positive cells in the injured area of tibialis anterior muscle 3 days after injury, few PAX7- and Ki67- double positive cells were observed in the equivalent area of *Cdk1sc*− mice (Figure 4B). Furthermore, the number of PAX7-positive satellite cells was lower in *Cdk1sc*− mice compared to *Cdk1sc+* mice at both 3 and 14 days after muscle injury (Figure 4B and Supplementary Figure S1). On the other hand, TUNEL assays failed to reveal any difference in apoptotic cells between *Cdk1sc*− mice and *Cdk1sc+* mice (Figure 4C). Therefore, the impaired recovery after muscle injury in *Cdk1sc*− mice is mainly caused by impaired proliferation but not apoptosis of muscle satellite cells.

### Satellite Cell-Specific *Cdk1*-Knockout Mice Have Normal Muscle Mass

**FIGURE 5:**
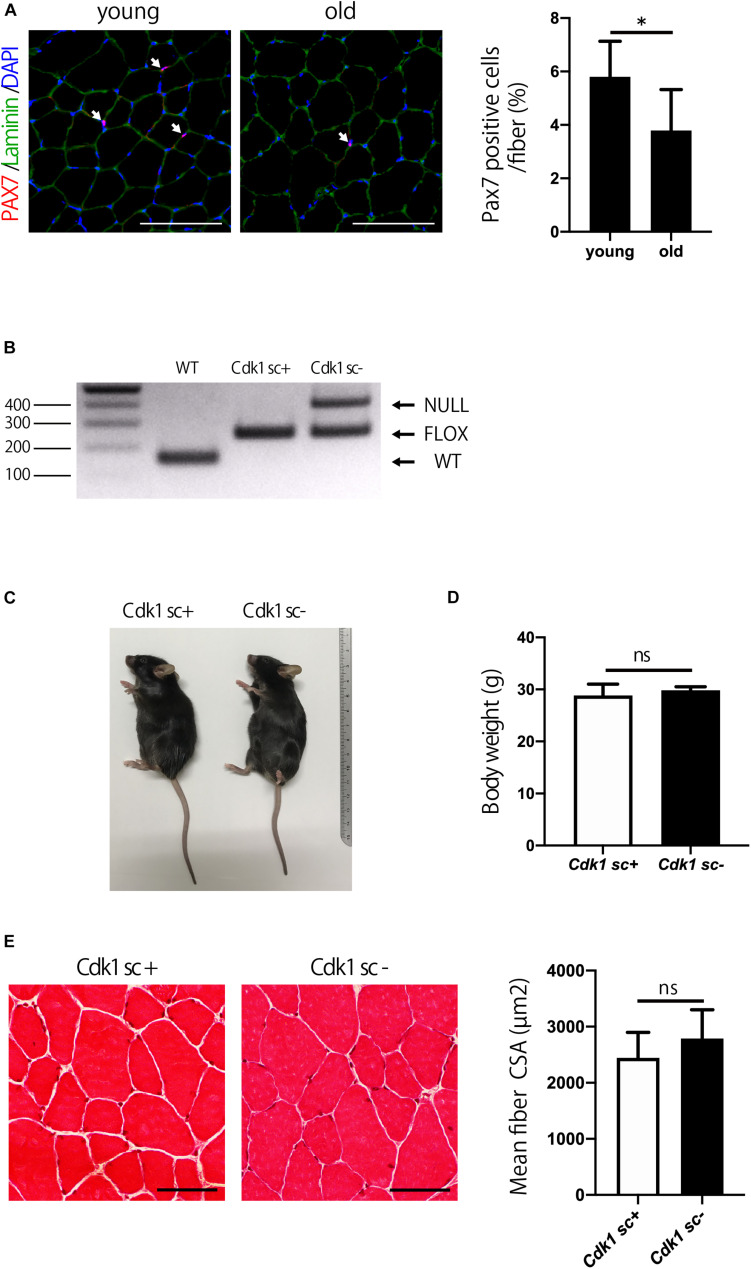
Satellite cell-specific *Cdk1*-knockout mice have normal muscle mass. **(A)** Immunohistochemistry analysis of laminin (green) and PAX7 (red) of mouse tibialis anterior muscles. DAPI (Blue) was used to stain the nuclei. These images show fewer PAX7 positive satellite cells (white arrows) in the old than in the young mice. Scale bars: 100 μm. ^∗^*P* < 0.05. **(B)** PCR genotyping demonstrated that the presence of null allele in the tibialis anterior muscle of *Cdk1sc*– mice. **(C)** The gross appearance of 1.5-year-old *Cdk1sc*– mice was not significantly altered in comparison to *Cdk1sc+* mice. **(D)** Bodyweight was comparable between *Cdk1sc*– and *Cdk1sc+* mice. n.s., not significant. **(E)** Histological analysis of the tibialis anterior of 1.5-year-old *Cdk1sc*– mice demonstrated no difference in cross-sectional area (CSA) compared with that of *Cdk1sc+* mice. n.s., not significant. Scale bars: 50 μm.

The number of satellite cells and muscle mass decline with aging (Demontis et al., 2013). Indeed, immunostaining of muscle sections showed that the number of PAX7 positive satellite cells was significantly reduced in 1.5-year-old (old) mice compared to 12-week-old (young) mice (Figure 5A). We then investigated the role of Cdk1 in the maintenance of muscle mass from adolescence to old age. To this end, 12-week-old female Pax7^CreER^/*Cdk1f/f* mice were treated with vehicle or tamoxifen by IP injection for 5 consecutive days to effectively eliminate Cdk1 in satellite cells and analyzed for muscle mass when mice reached 1.5 years of age. To investigate whether the deletion of *Cdk1* was maintained for 60 weeks after treatment, we extracted DNA from the tibialis anterior muscle of 1.5-year-old *Cdk1sc+* and *Cdk1sc*− mice and subjected them to genotyping. The results indicated that *Cdk1* was deleted in PAX7-expressing cells in the tibialis anterior muscle 60 weeks after treatment with tamoxifen, because the null allele was found in the muscle tissue of *Cdk1sc*− mice (Figure 5B). The additional visible floxed *Cdk1* allele is most likely due to contamination of PAX7- cells. Gross appearance (Figure 5C) and body weight (Figure 5D) of 1.5-year-old *Cdk1sc*− mice was comparable to *Cdk1sc+* mice. Histological analysis of the tibialis anterior of 1.5-year-old *Cdk1sc*− demonstrated no difference in CSA compared with that of *Cdk1sc+* mice (Figure 5E). Collectively, these results suggest that *Cdk1* is dispensable for muscle mass maintenance during aging—at least when it is deleted in 12-week-old mice.

### Cdk1 Is Important for Overload Hypertrophy

**FIGURE 6:**
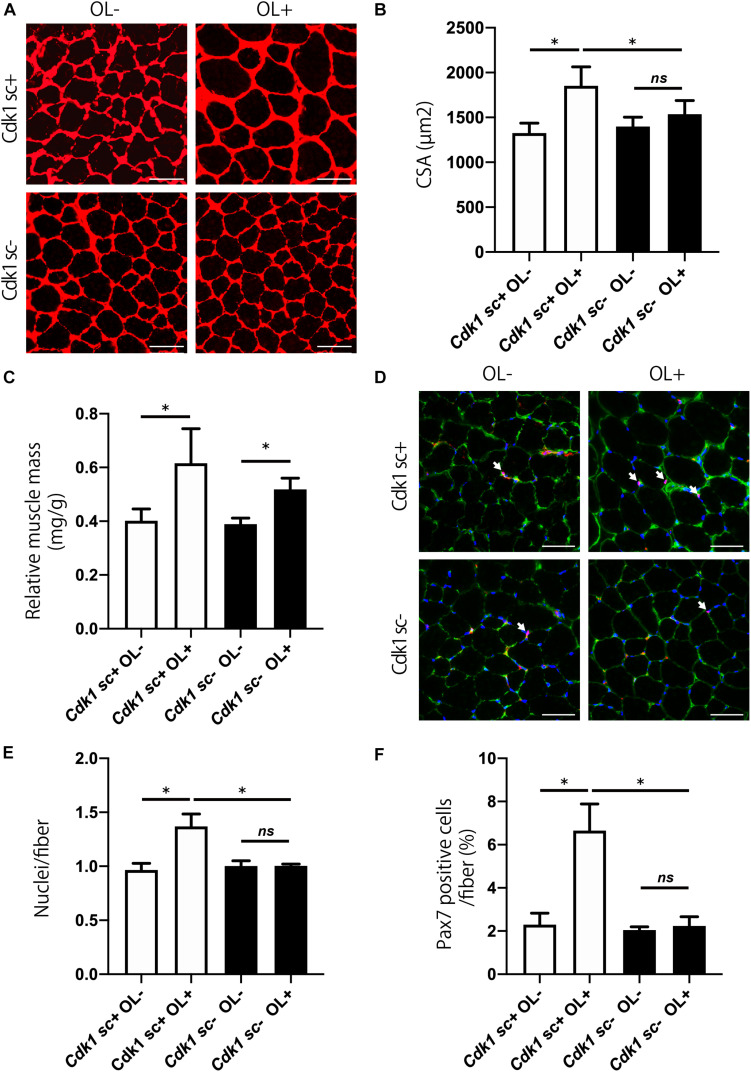
Genetic loss of *Cdk1* in satellite cells results in the absence of muscle hypertrophy. **(A)** Representative laminin-stained extensor digitorum longus sections depicting an increase in myofiber size after muscle overload in *Cdk1sc+* mice but not in *Cdk1sc*– mice. Scale bars: 50 μm. **(B)** Quantification of cross-sectional area (CSA) of myofibers at the extensor digitorum longus mid-belly reveals a significant inhibition of myofiber hypertrophy with satellite cell ablation of *Cdk1*. ^∗^*P* < 0.05. n.s., not significant. **(C)** Muscle weight of the extensor digitorum longus relative to body weight. Muscle weight increased by 53% in *Cdk1sc+* mice and 38% in *Cdk1sc*– mice by overload surgery. ^∗^*P* < 0.05. **(D)** Representative PAX7 (red)-stained extensor digitorum longus sections. DAPI (blue) was used to stain the nuclei. These images show fewer PAX7 positive satellite cells (white arrows) in *Cdk1sc*– mice than in *Cdk1sc+* mice by overload. Scale bars: 50 μm. **(E)** Quantification of the number of myonuclei at the extensor digitorum longus mid-belly reveals an increase in myonuclei number after muscle overload in *Cdk1sc+* mice but not in *Cdk1sc*– mice. ^∗^*P* < 0.05. n.s., not significant. **(F)** Quantification of the number of PAX7 positive satellite cells at the extensor digitorum longus mid-belly reveals an increase in satellite cell number after muscle overload in *Cdk1sc+* mice but not in *Cdk1sc*– mice. ^∗^*P* < 0.05. n.s., not significant.

We aimed to study the involvement of Cdk1 in muscle fiber hypertrophy, because the mechanism of muscle fiber hypertrophy is still unclear. To this end, we tested whether satellite cell-specific *Cdk1* deletion would affect the overload hypertrophy. While the overload surgery led to an increase in extensor digitorum longus mass in control mice, there was no significant difference in *Cdk1sc*− mice (Figure 6A). Histological observations revealed that overload led to a 49% increase in CSA in the extensor digitorum longus in *Cdk1sc+* mice. However, overload had less effect on average CSA in *Cdk1sc*− mice (Figure 6B). Muscle weight increased by 53% in *Cdk1sc+* mice and 38% in *Cdk1sc*− mice by overload surgery (Figure 6C). To determine whether the impaired muscle fiber hypertrophy in *Cdk1sc*− mice by overload is due to impaired satellite cell proliferation, we performed immunohistochemistry for PAX7 (Figure 6D). Overload increased the number of myonuclei in *Cdk1sc+* mice by 38%, whereas overload did not increase the number of myonuclei in *Cdk1sc*− mice (Figure 6E). Furthermore, Overload increased the number of satellite cells in *Cdk1sc+* mice by 189%, whereas overload did not increase the number of satellite cells in *Cdk1sc*− mice (Figure 6F). Collectively, our results suggested that *Cdk1* expression in muscle satellite cells, i.e., satellite cell proliferation, is vital for overload muscle fiber hypertrophy.

## Discussion

In this study, we investigated the regulatory roles of CDKs in satellite cell proliferation and regeneration. First, we showed that Cdk1 is expressed in myoblasts, and its mRNA expression and protein levels were down-regulated during myoblast differentiation. Then, we generated satellite cell-specific *Cdk1* knockout mice, which are viable. While the gross appearance of satellite cell-specific *Cdk1* knockout mice was normal, the deletion of *Cdk1* in satellite cells leads to impairment of muscle regeneration. Finally, we found that overload muscle fiber hypertrophy was Cdk1-dependent, at least in part. This study is, as far as we are aware, the first to demonstrate through *in vivo* loss-of-function experiments that Cdk1 has an important role in muscle regeneration.

With advancing age, the number and function of satellite cells are decreasing (Verdijk et al., 2014). However, it is controversial that whether age related deterioration of satellite cell proliferation occurs due to the extrinsic or intrinsic factors (Blau et al., 2015). The regenerative function of muscle satellite cells is regulated by their interaction with components of their extrinsic tissue microenvironment including systemic proteins and localized factors (Yin et al., 2013). In contrast, a recent report found that p16^INK4a^ was increased in satellite cells from aged mice and this was associated with deterioration of cell proliferation (Sousa-Victor et al., 2014). In our study, we found that decreasing Cdk1 expression in satellite cells led to deterioration in satellite cell proliferation and inhibition of muscle regeneration. Collectively, the results of this study suggested that not only extrinsic factors but also intrinsic factors in satellite cells might be associated with age-related deterioration in satellite cell proliferation.

We found that *Cdk1* deletion leads to deterioration in recovery after muscle injury. Generally, cell proliferation is considered to be a driving force in tissue repair (Krafts, 2010). However, for some tissues, this idea does not hold true. For example, even when hepatocyte proliferation is inhibited after hepatectomy, liver regeneration is compensated by the mechanism of cellular hypertrophy (Caldez et al., 2018, 2020). Interestingly, in bone tissue, parathyroid hormone treatment reverses bone loss after ovariectomy even when osteoblasts cannot proliferate (Takahashi et al., 2018). When muscle injury occurs, satellite cells proliferate extensively. Some of the progeny of these cells then fuse with each other to form replacement myofibers (Adams, 2006). On the other hand, in response to injury, activated satellite cells can also fuse with damaged but viable myofibers to promote repair and regeneration (Robertson et al., 1993; Adams, 2006). Hence, proliferation and fusion of satellite cells are required for proper muscle regeneration. However, with the previous methods of depleting satellite cells, it was unclear to what extent muscle recovery is compensated for by muscle fiber hypertrophy and cellular hypertrophy after muscle injury in a state where satellite cells are not depleted, as in the senescent state, but the proliferative capacity of the satellite cells is diminished. The results of our study suggest even if satellite cells are present, in situations where their proliferative capacity is reduced, recovery after muscle injury cannot be achieved by the compensatory mechanism of muscle fiber hypertrophy and cellular hypertrophy. Hence, these results indicate that Cdk1 is essential for satellite cell proliferation and muscle regeneration *in vivo*. Our results confirmed the notion that satellite cell proliferation is indispensable for recovery after muscle injury through *in vivo* cell-specific loss-of-function experiments.

Satellite cells are activated during periods of significantly increased muscle loading and some of these cells fuse with undamaged myofibers as part of the hypertrophy process (Adams, 2006). However, it is still controversial whether muscle fiber hypertrophy is dependent on satellite cell proliferation. So far, there have been several studies claiming that the muscle fiber hypertrophy was inhibited by radiation-induced satellite cell depletion and genetic satellite cell depletion (Rosenblatt et al., 1994; Adams, 2006; Egner et al., 2016). These results suggest that muscle fiber hypertrophy is dictated by the number of myonuclei present. However, another study showed that there was little difference in the muscle mass and fiber cross-sectional area associated with hypertrophy in the satellite cell-depleted mice compared with controls (Mccarthy et al., 2011). A human study also showed that moderate changes in skeletal muscle fiber size could be achieved without the addition of new myonuclei (Kadi et al., 2004). The results of our study indicated that although overload led to a 49% increase in CSA in the extensor digitorum longus in control mice, overload had no effect on average CSA in *Cdk1sc*− mice. Furthermore, overload significantly increased the number of myonuclei and satellite cells in Cdk1sc+ mice, but no such effect was observed in *Cdk1sc*− mice. These findings suggest that overload muscle fiber hypertrophy is dependent on satellite cell proliferation potential, thus supporting the former thesis rather than the latter. It should be noted that studies in which satellite cell-removed muscles showed an increase in fiber CSA with an increase in muscle mass and hypertrophy similar to that observed in the vehicle-treated group used plantaris (Mccarthy et al., 2011), but our study used extensor digitorum longus, and the genetic background of the mice used was different. On the other hand, muscle weight was significantly increased in *Cdk1sc*− mice, although to a lesser extent than in *Cdk1sc−* mice, due to overload. These results are similar to those of Egner et al. (2016). As Egner et al. (2016) stated that muscle weight is unreliable measure of hypertrophy, it may be difficult to remove the effect of surgical adhesions when looking at the effect of overload surgery on muscle weight. Interestingly, a recent study suggests that the mechanism of satellite cell proliferation was different between overloaded and regenerating muscles (Fukuda et al., 2019). We showed that in the absence of Cdk1, muscle hypertrophy in overload and muscle regeneration after injury was severely impaired, indicating that the expression of Cdk1 in satellite cells is vital in these processes.

Muscles are low-turnover tissues like the liver (Cheung and Rando, 2013). Interestingly, although impaired hepatocyte proliferation in the absence of Cdk1 was partially compensated for by hepatocyte hypertrophy after hepatectomy (Diril et al., 2012), the compensatory mechanism by muscle fiber hypertrophy did not take place when overload was applied in muscle tissue in this study. One finding explaining this mechanism may be that hepatocytes can reenter the cell cycle after hepatectomy, even after they have matured and differentiated (Fausto, 1997). Such a mechanism has not been previously reported in satellite cells *in vivo*. Based on our research, to establish therapeutic strategies to promote muscle regeneration and muscle fiber hypertrophy more effectively, it may be desirable to develop drugs that specifically enhance the proliferative capacity of satellite cells, or to cultivate and transplant strongly proliferating satellite cells.

Our study has several limitations. First, to determine the efficiency of Cdk1 deletion by Pax7^CreER^, a comparison of Cdk1 expression by isolation of satellite cells should be made. Unfortunately, we were unable to establish satellite cell cultures probably due to the reduced cell proliferation capacity caused by the deletion of Cdk1. Instead of isolating satellite cells, we used immunostaining and genotyping to demonstrate that the expression of Cdk1 was reduced in the PAX7-positive satellite cells in muscle tissue of *Cdk1sc*− mice. Second, to prove that Cdk1 is deleted in satellite cells of *Cdk1sc*− mice at age 1.5 years, ideally, immunostaining should be used to prove that CDK1 expression is down-regulated in satellite cells. But it was technically difficult to verify CDK1 expression in the static state using immunohistochemistry because CDK1 is only expressed during cell division (Ravnik and Wolgemuth, 1999) and skeletal muscle is a stable tissue that undergoes little turnover of nuclei (Stephenson and Kojouharov, 2018). Accordingly, to confirm that Cdk1 was deleted in satellite cells even after 60 weeks of tamoxifen treatment, we performed genotyping instead of immunostaining. PCR genotyping demonstrated that the presence of null allele in the tibialis anterior muscle of *Cdk1sc*− mice 60 weeks after tamoxifen treatment. These results suggest that in our *Cdk1sc*− mice, tamoxifen treatment resulted in a loss of Cdk1 in satellite cells from 12 weeks to 1.5 years of age.

## Conclusion

In conclusion, our data indicate that Cdk1 is required for satellite cell proliferation, muscle regeneration, and overload muscle fiber hypertrophy. From a clinical point of view, these findings could help to develop treatments for refractory muscle injuries and muscle atrophy, such as sarcopenia.

## Data Availability Statement

The raw data supporting the conclusions of this article will be made available by the authors, without undue reservation, to any qualified researcher.

## Ethics Statement

The animal study was reviewed and approved by the Animal Study Committee of Tokyo Medical and Dental University.

## Author Contributions

SS, PK, AO, and HI designed the study. YK, TT, MM, and HO performed the study. YK, TY, and HI analyzed the data. PK and HI wrote the manuscript. All authors contributed to the article and approved the submitted version.

## Conflict of Interest

PK is a specialty chief editor of this journal. The remaining authors declare that the research was conducted in the absence of any commercial or financial relationships that could be construed as a potential conflict of interest.
