# Supplementary material for: Cyclin-Dependent Kinase 1 Is Essential for Muscle Regeneration and Overload Muscle Fiber Hypertrophy
Source: Front Cell Dev Biol. 2020 Oct 14;8:564581. doi: 10.3389/fcell.2020.564581 (PMC7591635; doi:10.3389/fcell.2020.564581)
Supplement: Supplementary file 1 [file Table_1.DOCX]

Table S1. Primers for PCR genotyping for Pax7 and Cdk1

| Primer name | Primer sequence (5’- 3’) |
| --- | --- |
| Pr1 for Pax7 | CTGTGCTGG GACTTCTTCCT |
| Pr2 for Pax7 | AAAGACGGCAATATGGTGGA |
| Pr3 for Pax7 | AGACTCAGGGCTTGGGAAGG |
| Pr1 for Cdk1 | TTTGTCAAGAAATGCAGCAGGACAC |
| Pr2 for Cdk1 | GCTCACATGTTCCTGAATGGTGGA |
| Pr3 for Cdk1 | GCGGTGGTTTAAACCCATAATCCCGGCAC |

Table S2. Details of primers for qPCR

| Gene name | Primer sequence (5’- 3’) |
| --- | --- |
| Gapdh forward | AGGTCGGTGTGAACGGATTTG |
| Gapdh reverse | TGTAGACCATGTAGTTGAGGTCA |
| Cdk1 forward | GGAGTGCCCAGTACTGCAAT |
| Cdk1 reverse | TCTCTGTGAAGAACTCGCCG |
| Cdk2 forward | CCTGCTTATCAATGCAGAGGG |
| Cdk2 reverse | GTGCTGGGTACACACTAGGTG |
| Cdk4 forward | AAGGTCACCCTAGTGTTTGAGC |
| Cdk4 reverse | CCGCTTAGAAACTGACGCATTAG |
| Cdk6 forward | GGCGTACCCACAGAAACCATA |
| Cdk6 reverse | AGGTAAGGGCCATCTGAAAACT |
| MyoD forward | TGATGGCATGATGGATTACAGC |
| MyoD reverse | TGCAGTCGATCTCTCAAAGCAC |
| Myogenin forward | CATCCAGTACATTGAGCGCCTAC |
| Myogenin reverse | CTTAAAAGCCCCCTGCTACAGAA |
| Mrf4 forward | GGCTGGATCAGCAAGAGAAG |
| Mrf4 reverse | AAGAAAGGCGCTGAAGACTG |
