# Supplementary material for: Cyclin-Dependent Kinase 1 Is Essential for Muscle Regeneration and Overload Muscle Fiber Hypertrophy
Source: Front Cell Dev Biol. 2020 Oct 14;8:564581. doi: 10.3389/fcell.2020.564581 (PMC7591635; doi:10.3389/fcell.2020.564581)
Supplement: Supplementary file 2 [file Image_1.pdf]

Supplemental Figure 1

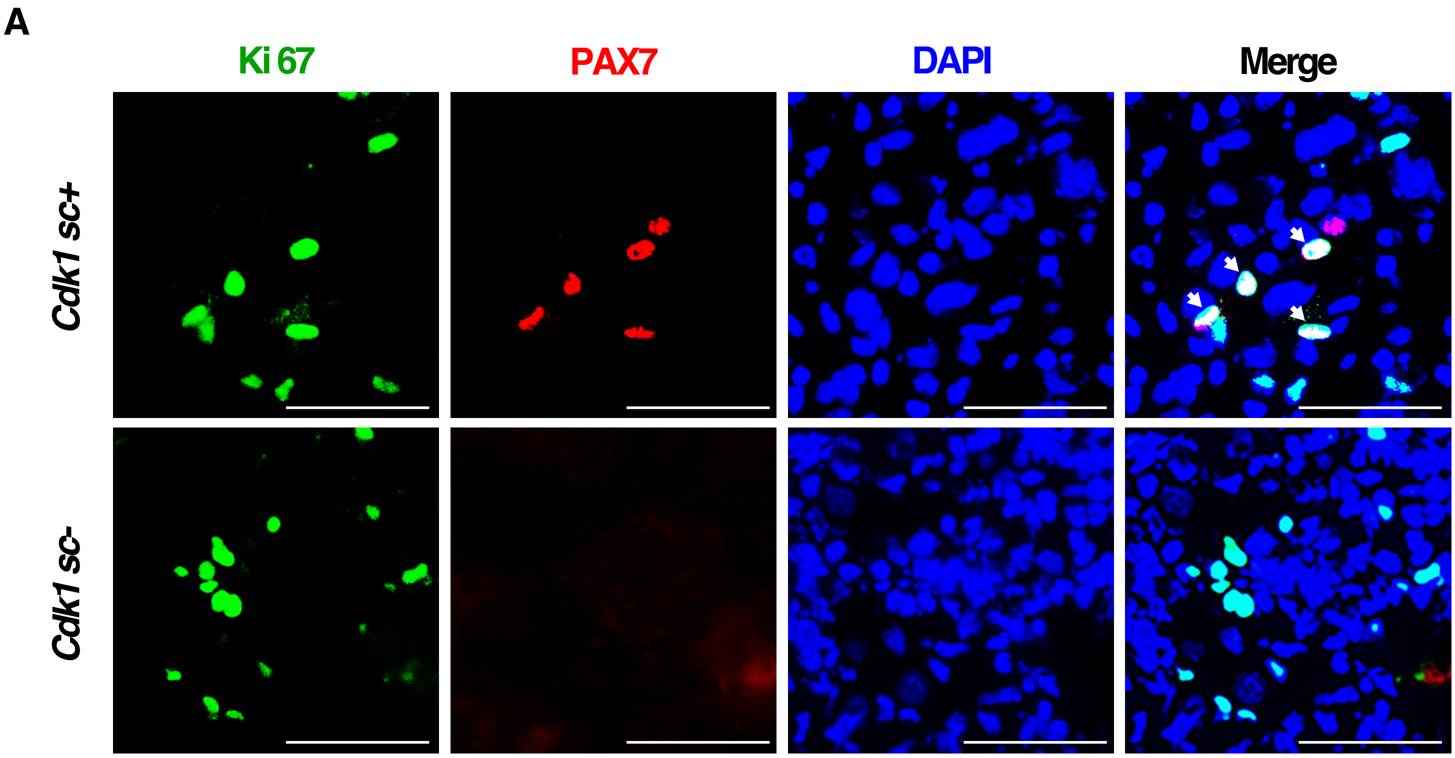

Supplemental Figure 1.
Colocalization of PAX7 with Ki67 in satellite cells. Immunohistochemistry to detect PAX7 and Ki67 in muscle tissue: Tibialis anterior muscle cryosections 3 days after cardiotoxin injection. PAX7 localization (Left, red), Ki67 localization (Middle left, green), DAPI localization (Middle right, blue), and merged image of green, red, and blue fluorescence (Right, white). Note the co-localization of PAX7 and Ki67 expression in satellite cells (arrowheads). Scale bars: 50μm.
